# Open-endedness in synthetic biology: A route to continual innovation for biological design

**DOI:** 10.1126/sciadv.adi3621

**Published:** 2024-01-19

**Authors:** Michiel Stock, Thomas E. Gorochowski

**Affiliations:** ^1^ KERMIT & Biobix, Department of Data Analysis and Mathematical Modelling, Ghent University, Ghent, Belgium.; ^2^ School of Biological Sciences, University of Bristol, Life Sciences Building, 24 Tyndall Avenue, Bristol BS8 1TQ, UK.; ^3^ BrisEngBio, School of Chemistry, University of Bristol, Cantock’s Close, Bristol BS8 1TS, UK.

## Abstract

Design in synthetic biology is typically goal oriented, aiming to repurpose or optimize existing biological functions, augmenting biology with new-to-nature capabilities, or creating life-like systems from scratch. While the field has seen many advances, bottlenecks in the complexity of the systems built are emerging and designs that function in the lab often fail when used in real-world contexts. Here, we propose an open-ended approach to biological design, with the novelty of designed biology being at least as important as how well it fulfils its goal. Rather than solely focusing on optimization toward a single best design, designing with novelty in mind may allow us to move beyond the diminishing returns we see in performance for most engineered biology. Research from the artificial life community has demonstrated that embracing novelty can automatically generate innovative and unexpected solutions to challenging problems beyond local optima. Synthetic biology offers the ideal playground to explore more creative approaches to biological design.

## Teaser

Designing biology with novelty in mind can lead to a more open-ended synthetic biology, with constant improvement and innovations.

## INTRODUCTION

Consider a fictitious biotech company called “Biloba,” which aims to change the world through pioneering applications of synthetic biology. Each year, they hold an annual keynote in which the CEO amazes audiences with numerous ground-breaking pieces of biotechnology: a virus-based battery, a plastic-degrading enzyme thousands of times more efficient than before, and a microbe capable of producing the most extraordinary new fragrances. When questioned about Biloba’s design process to conjure these marvels, the CEO smiles and states “my team merely follows their curiosity.” What would it take to implement a genuinely open-ended innovation pipeline like this for synthetic biology? What are the ingredients that are needed?

The natural world demonstrates that open-ended innovation is possible and pervasive. Darwin famously wrote about “endless forms most beautiful,” and biological evolution is considered open-ended (*1*). Nature continuously innovates, creating novel proteins, metabolisms, and morphologies to increase reproductive success, adapt to changing environments, and respond to other species through coevolutionary forces that often cause increases in complexity.

Many sociotechnical systems can also be considered open-ended. Technological innovations often advance so rapidly that making meaningful predictions about how an area might change only a few years into the future is impossible. This stems from the fact that recent technological advancements are often the basis for future innovations, making long-term predictions difficult (*2*). Similarly, the cultural sphere is a never-ending stream of stories and art with endless genres, movements, and new media. It is postulated that imagination is only limited by the number of concepts one can process and recombine (*3*, *4*).

Humans acting with varying degrees of control have harnessed biology in new and valuable ways for millennia, leading to the domestication of animals, plants, and microorganisms for food, medicine, and labor (*5*). Recent advances in synthetic biology have shown that more precise and elaborate engineering of living systems is also possible. This has opened the opportunity for biology itself to act as a basis for technological innovation. The major difference from historical approaches to biological modification is our newfound ability to precisely alter or augment biology in useful ways, leaning on our growing knowledge of how biology works across scales and advances in DNA sequencing and synthesis (*6*, *7*). When paired with an engineering mindset that brings together concepts of abstraction, standardization, and modularity (*8*), synthetic biology has been able to move beyond merely harnessing the inherent capabilities of biological systems and has pushed us toward creating entirely new-to-nature biological parts, devices, and systems (*9*, *10*).

Biological engineering is distinct from other more established engineering disciplines because biology can adapt and evolve (*11*). Typically, human-engineered machines are designed according to a “blueprint” and consist of modular parts that can be studied using a reductionist framework. In contrast, biological systems have evolved and arise through self-organizing structures that adapt to changing contexts and environments. Living systems are also characterized by emerging multiscale structures that are complex and robust to change, the human brain being perhaps the best example. Furthermore, human-made machines often have a hardware-software duality where functions can be programmed and interrupted. Biological systems lack this separation, having their function and information processing embodied as part of the organism itself. Information is encoded not only in an organism’s genome but also in the array of diverse components and processes making up each cell (*12*), e.g., the expressed proteins, metabolic fluxes, and cell membrane organization.

The ability for biology to evolve, i.e., its evolvability, can be both a blessing and a curse for synthetic biology. Evolution is a powerful engineering tool (*13*, *14*). However, it also allows biology to alter itself after deployment in unanticipated, potentially undesired, or dangerous ways (*15*–*17*). Directed evolution has become a routine tool in biotechnology for optimizing the function of biological components or systems (*13*, *18*–*20*). Typically, a bioengineer creates a set of variants via random or directed mutagenesis or combinatorial recombination methods like SCRaMbLE (*21*). The library of variants is then screened via a selection procedure that retains the most performant designs. These variants are then used as the starting point for further rounds of mutagenesis. While this artificial process captures some aspects of evolution (i.e., variation and selection), recent work has advocated for a broader and more informed integration of evolution in synthetic biology. Castle *et al.* (*14*) go a step further and consider the evolutionary landscape as something to be sculpted as part of the design process such that resultant biological designs are more robust or more evolvable when deployed: the engineering of evolution itself.

**Fig. 1.**
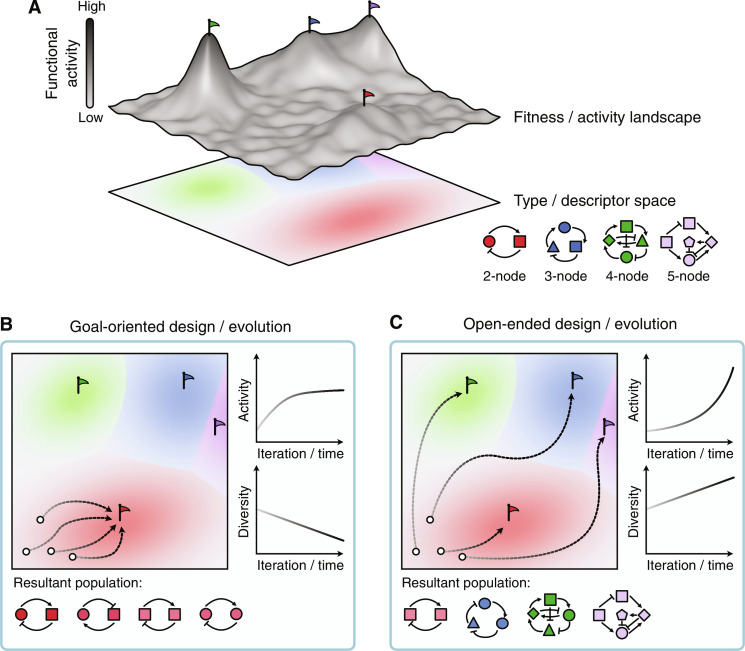
Goal-oriented versus open-ended design/evolution. (**A**) Each entity or construct can be represented as a point on a design landscape where distance corresponds to the degree of difference between designs. Each entity has a certain fitness or functional activity, shown by the height of the landscape at that point. Underneath is a type/descriptor landscape, indicated by a hue, that characterizes how the entities differ from each other (e.g., levels of complexity in terms of the number of components/nodes present). Flags represent optima for different “types” of entity (i.e., varying levels of complexity). (**B**) Traditional goal-based processes focus on improvement alone, thereby reducing the population’s diversity and leading to diminishing returns in improvement as local optima are reached. (**C**) Open-ended processes emphasize the continuous creation of novel entities, offering the opportunity to find new types of solution to a problem and greater opportunities for surprising solutions over time.

Starting from an evolutionary mindset, we provide a perspective on biological design that recognizes the crucial role that open-endedness and novelty play in biological evolution and their potential for integration into design workflows for synthetic biology (Fig. 1). Building on efforts by artificial life and evolutionary biology communities, we aim to demonstrate why a search for novelty, rather than optimizing function alone, is a more productive strategy for achieving continuous innovation. We want to persuade biological engineers that new approaches are required and to embrace creativity when designing the engineered biological systems of the future. Because of the breadth of topics covered, Box 1 provides a glossary of terms used throughout.

### WHAT IS OPEN-ENDEDNESS?

Open-endedness, or open-ended search, relates to the capacity of a system to endlessly improve, produce novelty, or increase its complexity over time (*22*–*24*). Open-ended processes are also sometimes characterized as having no clearly defined objective or goal (*25*). The degree of open-endedness can be formally characterized by methods of statistical and algorithmic information theory (*26*, *27*). Natural evolution, a process without a clearly defined goal, is a prime example of an open-ended process, giving rise to an endless diversity of organisms adapted to different environments and niches. Several human activities, such as technological innovation, the scientific process, and the creation of art and fiction, are also considered open-ended.

Open-endedness as a research topic emerged from the artificial life community. Artificial life researchers aim to capture some of the complexity of life via computer simulations, robotics, and the use of chemical and physical systems (*28*, *29*). The nature of open-endedness is a major open problem in artificial life (*30*) and a driving force behind its more fundamental research (*31*, *32*). Chemists, for instance, have recently explored abiogenesis models by trying to design chemical systems that would permit open-ended evolution (*33*). Open-endedness may also be one of the great frontiers of artificial intelligence (AI) (*24*, *34*–*36*) because it could act as a foundation for new technological innovations. The principles behind open-endedness have been explored in domains as diverse as developing new computer architectures (*37*), robotics (*38*–*42*), software design (*43*), artificial neural networks (*35*, *44*, *45*), and even cancer treatments (*46*). Although great strides have been made in our understanding, we are still far from replicating the open-endedness we see in natural systems.

The open-ended nature of biological evolution is well established. Bedau *et al.* (*47*) classified long-term evolutionary processes based on fossil records considering whether adaption was absent (no generation of new adaptations), bounded (generation of new adaptations but with a limited amount of observed diversity), or unbounded (generations of new adaptations with no seen limit to diversity) and found that diversity is a requirement for unbounded evolution. More recently, Vladar *et al.* (*1*) considered three models of evolution, depending on the evolutionary dynamics of a population. When in stasis (e.g., a well-adapted bacterial population), the system does not change, and the fitness stagnates. In a nonprogressive setting, different types of evolution continuously and recurrently appear as periodic fluctuations. Such cycles can arise due to coevolutionary effects like host-parasite arms races. Conversely, when the system is open-ended, it continuously changes in a subtle and structured way, giving rise to new evolvable traits. Complex microbial ecosystems in changing environments often exhibit such open-ended regimes.

Box 1 **Glossary of terms.**

• **Open-ended process**: A process that continually generates novel entities or entities that can improve their functionality without bound.

• **Designing**: Realizing an entity with certain specifications using a deliberate, top-down, goal-oriented plan.

• **Evolving**: Realizing an entity with certain specifications according to several iterations of random, bottom-up steps.

• **Evolvability**: The degree to which an entity can adapt through evolution.

• **Entity**: An integrated whole in the system. Entities can be composed of lower-level entities and might themselves be part of higher-order entities.

• **Design space**: The space of all possible configurations of the entities, similar to the genotype space, e.g., all possible amino acid sequences of a protein family.

• **Type/descriptor space**: The space of all possible arrangements and interactions between a system’s components, capturing the different types of system that are possible, e.g., all different folds of a protein family.

• **Emergent behavior**: Behavior or properties that arise from the interactions of underlying components of the entities but which cannot be directly deduced or predicted from the characteristics of those components in isolation.

• **Novelty**: The degree to which an entity is new or unusual, either due to novel compositions or by exhibiting new behaviors.

• **Variation**: Novelty that changes an entity by altering its internal arrangement of components.

• **Innovation**: Novelty that changes the entity by adding new kinds of components that can be used to build the entities.

• **Emergence**: Novelty that changes how the components interact.

• **Creativity**: The process of creating novelty.

• **Reality gap problem**: The observation that entities that are optimized for a goal in artificial environments (by simulation or in laboratory conditions) often do not perform this function well in realistic conditions because the simulator is an incomplete representation of reality.

• **Stepping stone problem**: The observation that to create an entity with a particular function, one often has to take intermediate steps that seem unrelated or counterproductive toward the end goal.

• **Optimization**: Branch of mathematics concerned with finding the best solutions to a given problem using an objective function, e.g., optimizing a metabolic pathway to maximize the yield of a desired small molecule.

• **Reinforcement learning**: Branch of artificial intelligence concerned with teaching agents to make optimal decisions to maximize a reward. The reward is typically tailored to the desired outcome, e.g., learning a strategy to select new strains for better biofuel production, where a reward is given based on the overall yield achieved to date.

• **Robustness**: The degree to which a system tolerates random perturbations and noise. A building block of evolvability as changes can accumulate without deteriorating functionality. Biological systems have numerous mechanisms to ensure robustness (*189*, *190*).

• **Modularity**: The degree to which a system is composed of roughly independent subsystems. A building block of evolvability because subsystems are more likely to be recombined into new, functional systems.

New traits or phenotypes arise from evolutionary innovation (*48*–*50*). These can be either performance innovations, which improve fitness but do not change the niche, or niche innovations, which create new opportunities for the species [e.g., allowing bacteria to grow on new substrates (*51*, *52*)]. Theoretical reasoning regarding computational complexity by Kaznatcheev (*53*) indicated that unbounded increases in fitness are possible in principle, and recent work elucidating empirical fitness landscapes has confirmed that wild-type RNAs (*54*) and proteins (*55*, *56*) are indeed suboptimal with room for improvements.

Regarding the composition of systems and features that enable open-endedness, several different terminologies are used throughout the literature. To be consistent, we will use a specific set of terminology established by Banzhaf *et al.* (*23*) where the units of interest (e.g., proteins, genetic constructs, and engineered cell) are called entities. Crucially, entities are a hierarchical concept and can be composed of lower-level entities (e.g., a genetic construct is composed of functional parts such as promoters, coding sequences, and terminators). At the same time, they may also form part of higher-level entities (e.g., several transcriptional units make up a metabolic pathway). The lowest level 0 entities are called atomic, and higher-level entities or abstractions become entities in their own right by the process of emergence (e.g., a genetic circuit can be abstracted as a logic gate or individual cells might form part of a multicellular organism).

Insights from algorithmic information theory (using the length of computer programs to study complexity) provide intriguing observations about the nature of atomic entities in open-ended systems. For example, building blocks are typically expected to follow the Zipf’s law (*27*), which states that the frequency of a building block being used is inversely proportional to its rank. This law states that a system’s 10th most-used building block will be used half as often as the fifth most-used block, and so on. Empirical evidence has confirmed the Zipf’s law in many open-ended systems, including word usage (*57*), evolved electronics (*58*), ingredient combinations (*59*), LEGO (*60*), and protein domains (*61*). This means that one can go a long way using a small number of general building blocks combined with some specific ones.

Entities form part of a wider environment, such as a flask, fermentor, cell, gut, etc., the combination of all entities and the environment is called the system. According to Witkowski and Ikegami (*62*), systems can become open-ended if they manifest (i) communication: there is constricted information flow between the entities and the environment (e.g., cells can exchange information through signalling); (ii) concurrency: the emergence of different temporal and spatial scales by concurrent stochastic processes (e.g., cells have compartments and organelles and biological processes such as enzymatic conversion or transcription take place at different time scales); and (iii) complexity: the system has the potential to grow in complexity (e.g., cells can acquire new functional elements in their genome).

Recent work has demonstrated a meaningful increase in complexity in simulated systems by allowing entities to organize in higher-level entities (*62*–*64*). In biological systems, the evolution of multicellularity (*65*) is a prime example of such a higher-level entity. The artificial transition to multicellularity has been realized in several experiments (*66*, *67*), while platforms like synNotch (*68*) and synCAM (*69*) have been used to let cells self-assemble into complex multicellular structures. Cells can also be assembled in a top-down fashion into completely new organisms with programmed behavior (*70*, *71*). Recent work showed that electron-conducting polymers could be used to develop artificial symbionts capable of autotrophically assimilating N_2_ and CO_2_ into γ-polyglutamic acid, attaining an efficiency far outstripping natural systems (*72*). Researchers have also implemented genetic circuits distributed over multiple cells, allowing scalability and flexibility in the circuits developed (*73*). Distributed genetic circuits like this can be used to control microbial consortia, for example, by density-depended expression of bacteriocins to overcome competitive exclusion (*74*). The theoretical principles of how entities assemble to form new, higher-level entities are currently an active area of research (*75*–*78*).

**Fig. 2.**
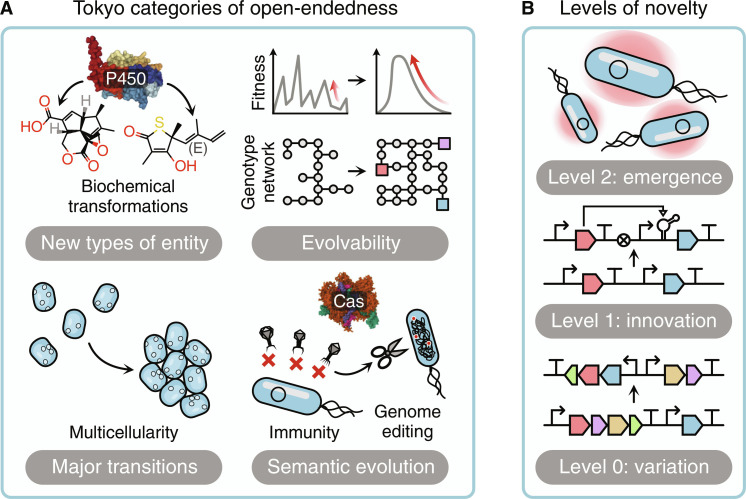
Overview of open-endedness and novelty. (**A**) The Tokyo categories of open-endedness. Open-ended systems generate unlimited (i) improvements and novelty (e.g., the promiscuity of P450 enzymes being modified toward new substrates), (ii) evolvability (i.e., entities changes so that they become more easily adaptable), (iii) major transitions (e.g., evolving yeasts to become multicellular), and (iv) semantic and functional evolution (e.g., the CRISPR-Cas system that changed from part of the bacterial immune system to become a tool for genome engineering). (**B**) Different levels of novelty, depending on the level of the entities it relates to. Level 0: Variations alter the entities without changing the basic toolkit (e.g., mutations in a genetic construct of a new arrangement). Level 1: Innovations extend the toolkit with novel building blocks without changing the concept of the toolkit (e.g., adding a new part for building genetic circuits). Level 2: Emergence changes how the model or toolkit operates (e.g., using the genetic circuit to create artificial quorum sensing systems, moving beyond the single bacterium).

Several efforts have been made to categorize the different types of open-endedness a system might exhibit. The York report (*79*) distinguishes either the ongoing generation of adaptive novelty (such as new adaptations, new entities, major transitions, and the evolution of evolvability) and an increase in complexity of both the entities and the interactions present. More recently, the revised Tokyo categories of open-ended evolution (Fig. 2A) (*31*) provide a clear set of hallmarks of open-endedness related to the ongoing generation of the following:

• New kinds of entities and interactions, where the system continuously produces novelty (e.g., P450 enzymes can be evolved to catalyze diverse kinds of reaction);

• Evolution of evolvability, where the entity changes such that it becomes easier to adapt (e.g., the evo-devo toolkit that makes morphological evolution easier to perform);

• Major transitions, characterized by emergent hierarchies (e.g., a transition from a single-celled to a multicellular form);

• Semantic evolution, where the functions of entities change [e.g., the CRISPR-Cas system evolved to act as a bacterial immune system but has since been repurposed by humans as a tool for genome engineering (*6*)].

### NOVELTY, INNOVATION, AND CREATIVITY

Open-endedness is closely tied to the generation of novelty. Novelty is the degree to which an entity is new or unusual and typically manifested in two ways. First, the composition of the entities might be new, roughly analog to a new genotype from existing parts (e.g., a new protein sequence from the same set of amino acids). Notably, one can assess this type of novelty before realizing (i.e., expressing) the physical entity. Second, novelty can relate to the behavior of the entities (i.e., the phenotype of the construct or organism), for example, a protein with an unusual fold enabling new catalytic capabilities. Recent advances in deep learning–assisted protein design “hallucinate” a wide diversity of protein scaffolds to engineer such as new-to-nature luciferases (*80*) or enzybiotics (*81*). Phenotypic novelty is usually more informative than genotypic novelty because it relates to the function or emergent properties of the system (*25*, *32*). However, this type of novelty can typically only be assessed after physically creating, simulating, or making predictions about the system as a whole. Novelty typically only makes sense in the context of a population of evolving entities or some historical record of genotypes/phenotypes that have been seen before.

Considering the framework developed by Banzhaf *et al.* (*23*), where entities are organized into different levels, we can classify different types of novelty in relation to the level it affects (Fig. 2B):

•Level 0: Variation—Novelty within the model that maintains the fundamental nature of the building blocks or types of connections. At this level, changes are made to the properties of one of the components, e.g., using a different promoter in a construct or adjusting the reaction rates within a metabolic network.

•Level 1: Innovation—Novelty by introducing a new kind of building block, adding a new dimension of variety. At this level, the combinatorics of the model are changed, increasing or decreasing the possibilities of variation, e.g., by allowing noncanonical amino acids in a protein sequence, introducing new kinds of parts for genetic constructs, or introducing new metabolic reactions that were not present before.

•Level 2: Emergence—Novelties that change the nature of the interactions between the building blocks, allowing higher-order interactions, e.g., multiprotein constructs such as tailocins, implementing quorum sensing to enable communication between cells, or harnessing the metabolic interactions within a community of microorganisms for enhanced productivity.

Taylor (*50*) presented a similar three-level hierarchy with differing terminology. In their case, exploratory novelty finds new configurations or behaviors within the system, expansive novelty extends the system with new concepts and functional parts, and transformational novelty extends the system itself.

Creativity, as defined by Boden (*82*), is the ability to generate entities that are new, unexpected, and potentially valuable (i.e., the process of generating novelty). Boden identifies three forms of creativity: (i) exploratory creativity involves the creation of new concepts within the conceptual space (variation), e.g., finding a new protein variant; (ii) combinatorial creativity, which involves novel connections between entities (innovation), which explores novel connections and combinations between existing concepts, e.g., inventing a new pathway; and (iii) transformational creativity, which changes the concept space itself (emergence), e.g., engineering with semisynthetic cells (*83*) that opens a new interface between living and nonliving components.

Artificial life researchers have historically been fascinated by computational creativity (*82*, *84*, *85*). Traditional evolutionary algorithms, like genetic algorithms, extensively use mutation and recombination to explore new solutions and concepts (*85*, *86*). At the same time, recent methods that use deep learning have also gained traction (*87*–*89*). Methods such as genetic programming, which search the space of possible functions and computer programs, are of a higher level of creativity, as they can expand the conceptual space by increasing the complexity of the functions present, thereby enabling transformational creativity. Recently, Lehman *et al.* (*90*) gave an extensive account of cases where artificial life simulations exhibited completely unexpected behaviors.

The fields of AI and machine learning have also explored the role of creativity and novelty in various contexts. For example, extensive work has been done on using deep learning for creativity (*88*), and the area of neuroevolution exploits evolutionary algorithms to uncover novel neural network building blocks, architectures, and learning algorithms that may one day help underpin artificial general intelligence (*91*).

### NOVELTY BEFORE FITNESS

The goal of directed evolution is usually to improve an entity’s fitness or functional performance (e.g., the catalytic rate of an enzyme). In evolutionary computing, fitness denotes the general quality or objective of the solutions, whereas in biology, the term is generally reserved for the reproductive success of an organism. Here, we use goal, fitness, or functionality to refer to how well an entity fulfils its desired function. The concept of a fitness landscape (*92*) is commonly used when considering such processes. Mutation and recombination randomly move entities around the landscape, while natural or artificial selection drives these movements toward higher fitness peaks. Although fitness landscapes are suited to thinking about improvements in function, they can be misleading when trying to obtain novel entities or functionalities. The reason for this is that fitness landscapes emphasize immediate short-term fitness improvements (i.e., taking small steps toward peaks of fitness) and do not allow reasoning about generating novel functions and phenotypes because novelty cannot be directly represented on a fitness landscape: It is a property of the population or the entity’s life history.

The artificial life community has identified two major issues with goal-oriented evolution, which are particularly relevant for synthetic biology: The reality gap problem, as fitness landscapes in artificial environments usually do not match reality and the stepping stone problem where endowing an entity with a particular function (e.g., engineering a bacterium to produce a novel metabolite) might require nonobvious or counterintuitive intermediate steps.

The reality gap problem highlights that entities evolved in simulated or artificial environments do not always work well in real-life situations (*41*, *90*). This problem stems from them being selected to function in a simplified computer model, allowing them to exploit the peculiarities of the simulator or causing them to be unable to deal with the vastly more complex reality in which they are deployed. Similar issues in biotechnology are numerous. Scaling bioremediation beyond the laboratory remains challenging due to the complexity of microbial communities in natural environments (*93*). Phage therapy is often less effective than expected due to the presence of biofilms and the interaction with the human immune system (*94*). The reality gap problem is a major challenge in synthetic biology, where systems are commonly developed in carefully controlled lab conditions, causing them to be fragile when deployed into highly variable real-world environments (*95*).

The stepping stone problem is the observation that to bestow an entity with a new function, the intermediate steps often only make sense retrospectively. Examples of the stepping stone problem are ubiquitous in technological innovation (*22*), for example, in the technological advancements needed to realize a computer (*96*). The existence of stepping stones was elegantly demonstrated by the PicBreeder project (*97*), a system that generates abstract images that users can “breed” to generate subjectively appealing images. Although people managed to create images of cars, skulls, and butterflies, the intermediate images of their lineages were often abstract. Goal-based search methods failed to discover such images even though they were present in the space (*22*, *41*). In biological evolution, birds are another good example: Feathers likely evolved for thermoregulation before being repurposed for flight (*98*). The stepping stone problem has important implications for synthetic biology, as developing new functions, such as producing a new-to-nature compound in a bacterium, might require several steps that do not advance or even hinder the target objective but are essential to realizing an optimal yield.

Current views of evolution balance selectionism (i.e., improving fitness) with neutralism (i.e., variation that does not impact fitness). Wagner (*48*) introduced genotype networks as an elegant framework for exploring evolutionary innovations that emphasize neutral processes and biological systems’ self-organization. Genotype networks are graphs containing all genotypes that encode the same phenotype and are connected by a single mutational step. Theoretical reasoning and empirical observations show that such networks are typically enormous and cover large parts of the genotype space. This allows entities to cover vast genetic distances using neutral processes. Furthermore, the genotype for different phenotypes is often heavily intertwined. Mutations enable the entities to randomly walk their respective genotype networks, being shielded from selective forces. This offers opportunities for novelty to emerge rapidly as beneficial phenotypes can be reached in a single step.

Numerous computational studies support the existence of genotype networks (*99*–*102*). Recently, strong empirical evidence for genotype networks has also been obtained for RNA (*103*) and gene regulatory networks (*104*). The topology of genotype networks has been found to impact evolutionary dynamics substantially (*105*). Several evolutionary frameworks such as constructive neutral evolution (*106*) and zero force evolutionary law (*107*) can explain how neutral processes can increase the complexity of entities, which can act as a feeding ground for novel innovations.

Although neutrality can promote exploration through random drift, there is a more effective approach when using artificial selection. Stanley and Lehman (*22*) put forward that novelty and curiosity are powerful drivers of innovation, both biologically and technologically. Experimentally, they show that a search for novelty can solve problems (e.g., evolving a robot to solve a complex maze) where traditional goal-based evolution fails (*108*–*111*). By building on new behaviors without using the target goal as a guideline, the entities are shown to solve the problem as a by-product. However, for this to work, care must be taken about how novelty is defined and aligned with the goal. In the case of evolving robots to solve mazes, robots that exhibit novel exploratory strategies were selected, which proved to be effective for such tasks (*25*). Quality-diversity algorithms (*109*), which we discuss later, use novelty and diversity together with an objective or fitness to great success.

Novelty-based search can alleviate both the reality gap and the stepping stone problem. The entities are less likely to fall into the trap of local optima of an objective function because the goal is not directly used (*112*). The novelties generated during the process can act as stepping stones toward the end goal. Similarly, by focusing on novelty or quality and diversity, many different types of solutions are obtained. This diversity makes it more likely that some of these will work in reality because various strategies or mechanisms will be used, limiting the chance of all solutions exploiting the peculiarities of the simulator or lab environment. This was illustrated using the PicBreeder project: Search strategies that use novelty are, in contrast to target-based search, capable of recovering specific target images (*112*). Novelty can, therefore, lift microevolution, which makes minor tweaks to improve fitness, to macroevolution, where great leaps in the evolutionary landscape are made toward entirely new evolutionary innovations. One explanation is that novelty search is sufficient to induce evolvability (*113*). By selecting novel entities, one necessarily selects entities that easily give rise to new phenotypes.

In optimization and reinforcement learning (the fields concerned with automatically finding optimal solutions and policies), there is the exploration versus exploitation trade-off (*114*), where a search algorithm has to decide between exploring new strategies or improving a known high-value strategy. A focus on novelty provides a solution to this trade-off (*115*). Novelty search when reaching a steady state ensures a uniform sampling of the reachable phenotypic space (*116*). In contrast, sampling the design or genotypic space (e.g., using random mutagenesis or recombination) may provide a broad view of genotypic space. However, this does not guarantee that the process will observe the diverse functionalities that are present in the more complex phenotypic space.

### REALIZING OPEN-ENDEDNESS FOR BIOLOGICAL DESIGN

**Fig. 3.**
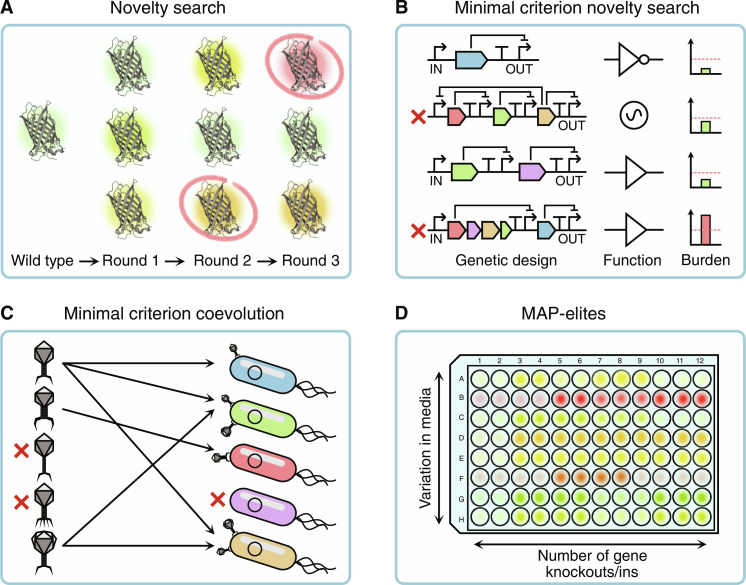
Overview of some important algorithms that can realize continuous improvement presented as hypothetical lab implementations. See main text for technical descriptions. (**A**) Novelty search: When using mutagenesis on fluorescent proteins, always retain the variants that emit spectra different from those encountered before, even if they are less bright. (**B**) Minimal criterion novelty search: When testing new genetic circuits, keep those that can compute new logic functions, provided they have minimal activity and are not trivial, i.e., the output depends on all inputs. (**C**) Minimal criterion coevolution: Phage training by coevolving the phages and bacterial host strains. Only retain for the next round the phages (bacteria) that can infect (be infected by) at least one bacteria (phage). (**D**) MAP-elites: in metabolic engineering, keep the best variant for all combinations of different media compositions and the number of knockouts or knock-ins performed.

Artificial life and evolutionary computing communities have recently made great strides toward realizing open-endedness, but how might we use these approaches for synthetic biology? This section outlines some key algorithms that can underpin open-endedness and ways they might be implemented in the lab (summarized in Fig. 3). When reproducing these algorithms, the degree to which the scientist is involved can vary greatly. The setup might be a “pot” in which new entities emerge or hands-on where a scientist manually characterizes and selects entities. Progress in simulation and machine learning [e.g., AlphaFold (*117*)] may also allow direct application of these algorithms in silico, further accelerating the process.

Generating random variation in the entities without selection is the most straightforward way of realizing open-endedness. However, it must be recognized that selective forces always affect living systems, as viability and growth rate affect the composition of populations over time. Here, we note that the bioengineer is not actively selecting for a particular functionality or trait. Randomness is a passive way of generating novelty, and it is a proven method for escaping local minima in mathematical optimization problems (*118*). Random mutagenesis is routinely used in biotechnology to obtain genetic diversity, particularly in plant breeding (*119*). In a similar vein, a decade-long microbiology experiment monitored 40,000 generations of *Escherichia coli* without actively selecting for a particular phenotype (*120*). Throughout these generations, the bacteria acquired the capacity to metabolize citrate as a new energy source (*51*, *52*).

To illustrate key algorithms in this area that more actively control aspects of an evolutionary search, let us consider a case where we want to obtain a fluorescent protein with a specific excitation-emission response (Fig. 3A). Typically, when using directed evolution, we would generate random diversity in the protein sequence and select variants closer to the response we need, carrying out the same mutation and selection cycle until a solution is found. An alternative, more open-ended approach is to screen for novel colours, regardless of the goal. This strategy makes it likely that the bioengineers encounter all kinds of new fluorescent proteins, including ones close to the desired colour (which could undergo a more targeted optimization). This type of approach has been used to find novel fluorescent proteins (*121*) and is an implementation of novelty search, which actively tries to find novel and unexpected entities (*25*, *108*). This method uses a novelty score rather than a fitness measure, which can be mathematically quantified by looking at the density in the type/descriptor space. The novelty score can either be based on the genotypic or phenotypic descriptors of the entities.

As a second example, consider the search for novel genetic circuits that can perform useful Boolean logic functions by modifying or rearranging basic genetic parts like promoters, coding regions, and terminators (Fig. 3B). We can use the logic functions a population can realize to calculate whether a circuit is novel or not. However, we immediately exclude oscillators and circuits that induce a considerable energetic burden that makes the cell close to non-viable. This type of protocol is a variant of minimal criterion novelty search (*122*), where the engineer performs novelty search yet has a specific criterion, such as a minimal activity, that the entities must satisfy.

Coevolution has also been shown to create diversity without needing to measure novelty explicitly (*123*, *124*); although pure competition does not always guarantee open-ended evolution (*1*). An example of this is phage training (i.e., making a wild-type phage better equipped to infect a specific bacterial strain) by coevolution where both host and phage can mutate (Fig. 3C). After every round, we only retain phages that can infect at least one bacterial strain and bacterial strains that can be infected by at least one phage variant. We purposefully are not too stringent in setting our goal, being able to infect a single bacterium or being infected by a single phage suffices, and there is no need to be optimal. This is an instance of minimal criterion coevolution (*125*). In this case, two interacting populations are maintained, with entities only having to meet some minimal functionality criterion to be included. Random variations introduced after each cycle ensure that the population’s diversity is maintained while the criterion ensures they remain functional. The paired open-ended trailblazer (POET) algorithm (*42*) is a more recent variant of this approach that actively tries to diversify the population by automatically generating environments (e.g., new substrates, hosts, or targets) to promote open-ended learning. POET has been shown to be capable of establishing open-endedness in reinforcement learning applications. Coevolution has also been successfully applied in biotechnology, for example, in phage training (*126*) and to induce multicellularity (*67*).

What if we explicitly want to improve the entities while still maintaining diversity across a population (i.e., reducing the change of diminishing returns)? Quality-diversity algorithms (*109*) still use an objective while demanding that the solutions are functionally diverse. A straightforward way to achieve this is to create niches based on genotype or phenotype and retain the best-performing entity within each niche, as opposed to some general overall performance (Fig. 3D). For example, suppose we wanted to engineer a microbe to break down a pollutant. We can distinguish variants between, for example, the number of genes that have been modified or by growing them in different substrate formulations. In each round, we would retain the best degraders for each number of genes targeted (hence, obtaining a collection varying in differences between the previous round) within each medium type. Such a protocol might be performed in a 96-well plate, ensuring various qualitative and quantitatively diverse solutions. This example is a lab implementation of the multidimensional archive of phenotypic (MAP)–elites algorithm (*127*), one of the most popular and successful quality-diversity algorithms. The MAP-elites algorithm has been found to be highly successful in evolutionary robotics (*128*). As the niches are clearly defined beforehand, MAP-elites is more stable than novelty search, where novelty changes throughout the run.

### INGREDIENTS FOR OPEN-ENDED EVOLUTION IN SYNTHETIC BIOSYSTEMS

A system is not necessarily endowed with open-endedness. Instead, it is a property that can be acquired (*129*). Iterative modification of the entities, combined with active or passive selection, leads to an evolutionary process. When selecting for functionality or higher fitness, the entity improves, although often with diminishing returns (*130*, *131*). At some point, the population stops changing while the diversity plummets (*132*). However, it is possible to design systems such that the entities themselves become more evolvable. In this section, we consider the conditions and processes bioengineers could implement directly within their systems to maximize the designed entities’ capacity to adapt, explore, and improve without bound.

Richard Dawkins coined the term “evolution of evolvability” to describe systems improving their capacity to evolve (e.g., by making it easier to reach beneficial phenotypes and harder to reach nefarious ones) (*133*). Evolvability can be directly selected for artificially (*134*, *135*). There are three building blocks of evolvability (*136*): (i) robustness, which quantifies how many changes the entity can accumulate while retaining its function; (ii) modularity, the degree to which the entities subcomponents can be recombined into new functional entities; and (iii) genotype-phenotype mapping, which links how changes in the entities’ genotypes impact their function. Each of these building blocks can be modified using numerous mechanisms through a process that has been called “evotype engineering” (*14*). Robust systems can easily be tinkered with and can accommodate many changes as neutral to fitness. This allows for broad exploration of the design space and discovery of novel phenotypes (*48*). The principle of modularity is also common in biology [e.g., in protein domains (*137*–*140*) and genetic regulatory/coding elements (*141*)] enabling building blocks to be recombined at will to create new designs. However, it has also been shown that in some cases, changes in the context of how parts are used (e.g., differences in nearby sequences of genetic parts) can substantially alter original functions (*142*–*147*). Last, genetic circuits and, more generally, biological computing (*148*–*150*) are potent tools of the bioengineer to control the expression of the phenotype based on internal and external information of the cell.

**Fig. 4.**
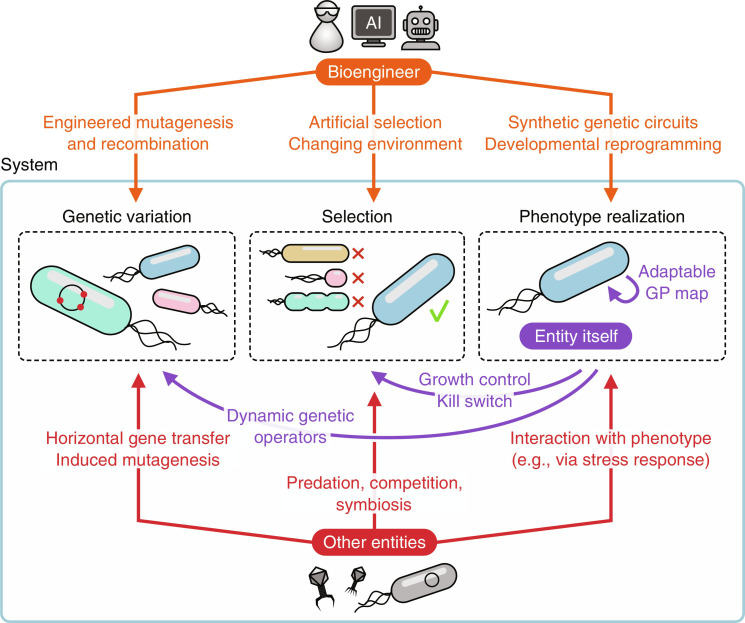
Routes to inducing open-ended evolution. Inspired by Taylor (*50*) to highlight potential interactions by a bioengineer that could be a human and/or AI system. Biological systems that can evolve have: (i) a system that induces genetic diversity, (ii) a selection system that determines which entities can reproduce or survive, and (iii) a genotype-phenotype mapping. Introducing feedback or perturbations to these systems, either externally in the entity or externally by the environment, another entity or the bioengineer ensures the system will not stagnate but keeps improving, changing, and evolving.

By taking a closer look at the different components of a system able to evolve, it is possible to devise strategies to elucidate the ingredients for open-ended evolution. The evolutionary trajectory of Darwinian evolution is determined by the evaluation of the phenotype (how it influences reproduction, either by natural or artificial selection) and genotype-phenotype map influenced by biotic and abiotic factors. By definition, only phenotypic realizations can influence the system. If these three aspects act in isolation, the entities improve toward a local optimum in the fitness landscape with diminishing returns. In relation to the novelty framework mentioned earlier, the systems create variation (level 0), but no innovations (level 1) or emergence (level 2) that extend and change the systems capabilities (*50*). To move beyond this, the system can be expanded by allowing for interactions between the three components, either internally by the components of the entity itself or externally by other entities, the environment, or the actions of the bioengineer. Figure 4 shows these components, together with interactions that bioengineers can create or manipulate.

The genotype-phenotype map of biological entities is naturally robust, modular, plastic, and, by consequence, evolvable (*151*–*153*). Gene regulatory networks control how the genotype is expressed based on internal and external states. When engineering organisms, genetic circuits allow immense flexibility in modifying phenotypic realization. The bioengineer can directly affect selection by artificially selecting for functionality, specific properties, or novelty. One simple yet powerful way to affect evolvability is by alternating the goal or environment, referred to as goal switching (*87*, *154*). This can be done, for example, by changing the type of growth medium of a microorganism or different conditions for optimizing an enzymatic reaction. Simulations on evolving circuits have shown that varying conditions substantially increased the modularity of the entities (*155*). Adding a connection cost to evolve networks results in more modular and eventually more evolvable and fitter networks (*156*). Similarly, coevolution has been shown to help avoid local optima (*123*, *157*). In biotechnology, coevolution has been used to evolve multicellularity versions of unicellular algae *Chlamydomonas reinhardtii* in a mere 750 generations (*158*). The selection process can also be directly engineered using biotechnological means, such as kill switches or dynamic growth control. Systems such as phage-assisted continuous evolution (PACE) allow the directed evolution of proteins in phages by an externally controlled selection (*159*). The functionality of a protein-of-interest encoded in a phage determines whether the bacterial host will express a pIII protein the phage needs to replicate new, infectious particles.

Last, in nature, genetic variation is far from a random process. Environmental stresses can induce highly localized hypermutation in bacteria, drastically improving the odds of beneficial changes (*160*, *161*). In enzyme engineering, by first learning the evolutionary constraints of chorismate mutase, recent work exploited specialized DNA synthesis to create many diverse enzyme homologs for directed evolution (*162*). Random and directed mutagenesis, error-prone replication, genetic engineering, or recombination using systems such as the SCRaMbLE platform (*21*) also allow for direct interventions in generating genetic variation. The generation of genetic variation itself can also be finely tuned by the bioengineer (*163*). OrthoRep is an orthogonal error-prone DNA polymerase for yeast (*164*), allowing chosen genes to mutate far beyond the error threshold for endogenous genes. Taking this a step further, genetic circuits have been used to implement feedback-regulated evolution, allowing the entity itself to change its mutation rate dynamically (*165*, *166*). PACE also illustrates how genes encoded in one organism, a phage, can be mutated by another organism, the bacterial host, while phage-mediated horizontal gene transfer is an example of phages that influence bacterial genetic variation.

### AN AMBITIOUS FUTURE

The design-build-test-learn (DBTL) cycle (*167*) has helped to establish synthetic biology as an engineering discipline. It combines a rational, knowledge-driven (design) and an empirical mindset (learn) with experimental validation (build and test) to deal with the uncertainty present when building living systems. The DBTL cycle is inherently goal-oriented. We invite synthetic biologists to think of their discipline as a creative one in addition to a technical one. Creating a superb culinary recipe requires expertise, but a master chef strives to create new culinary sensations. With this in mind, the synthetic biology community should consider exploring alternative paradigms to DBTL to uncover novel capacities of biological substrates. For instance, C-K theory (*168*) distinguishes a knowledge space in which statements are either true or false (e.g., promoter A induced by compound B), from a concept space, where so-called “crazy ideas” (e.g., a genetic circuit encoding a XAND logic function) can be explored. The concept space is freely expanded based on existing knowledge, while concepts encourage generating new knowledge (e.g., through experimentation). Such frameworks promote creative exploration, in addition to knowledge generation, and would allow biological engineers to move beyond mere problem-solving.

Novelty-based selection can be particularly effective in synthetic biology to engineer proteins, constructs, organisms, or communities. To implement such a strategy, an intelligent actor [e.g., the bioengineer or an AI (*169*)] is needed to judge the novelty by comparing it to the archive of characterized entities. Biological designs that would not be viable or active outside the lab can be protected and serve as a basis for new, more directly valuable designs.

Just as the available ingredients limit our ability to create new recipes, what we can build in the lab is similarly determined by the available biological components to hand. A massive number of different recipes can be created with just a limited number of ingredients (level 0). Discovering a new herb or protein allows the chef to expand their repertoire using innovations combinatorially (level 1), while further new preparation methods unlock the potential emergence of yet more options (level 2). In synthetic biology, we need to ask: which building blocks do we need and how can we best find them? Bioinformatics tools such as PartCrafter (*170*) and other metagenomics approaches (*171*) can aid in discovering biological components from novel sources, such as phage (*172*) or fungi (*173*) genomes. Others have used dictionary learning methods to identify reusable building blocks from biological systems (*174*). For instance, in protein engineering, recent work used machine learning to find protein modules with minimal epistatic effects one can freely recombine into novel enzymes (*140*).

The ability of living systems to evolve poses unique challenges for bioengineers, in particular in ensuring the reliability of functionalities achieved. Implementing evolution as a biodesign principle, such as enhancing an entity’s evolvability, also raises substantial concerns regarding biosecurity and unintended consequences. The entities developed through the methodologies presented in this work do not stem from deliberate methodical design, but instead arise from the exploration of a biological design space. This makes prediction and control of their behavior inherently challenging. Current recommendations propose limiting the use of evolutionary mechanisms to the design phase alone, with subsequent optimization of these evolved designs to mitigate safety concerns and limit future evolutionary paths (*15*). While traditional biosafety guidelines for synthetic biology offer a foundational basis (*175*, *176*), additional precautions, such as containment (*177*, *178*) or kill switches (*179*, *180*), are likely to be necessary to minimize the risk. Furthermore, enhanced screening methods are also likely to help reduce the chance of creating harmful agents unwittingly (*181*, *182*). Despite such measures, the potential for natural or engineered biological entities to reproduce and evolve beyond the intended control of bioengineers remains a notable challenge.

In pursuing the safe development of open-ended synthetic biology, we advocate drawing insights from AI safety literature, as exemplified in Bostrom’s “Superintelligence” (*183*). A key challenge in creating (semi-)autonomous AI agents is establishing sensible goals to avoid unintended consequences and facilitating safe exploration within a changing environment (*184*, *185*). Recent research considering open-ended approaches for realizing artificial general intelligence also emphasize safety concerns (*35*, *186*, *187*). The discrepancy between the bioengineer’s intended objectives (e.g., developing novel enzymatic variants), the explicit experimentally encoded incentives (e.g., selecting variants with differing substrate affinity), and the incentives of the evolving entity (e.g., reproduction and survival) present a significant challenge. Evolving entities often produce unexpected outcomes (*90*). Despite the challenges and potential risks associated with open-ended systems, managing them is not inherently impossible. Humanity has historically harnessed natural diversity through breeding and ecosystem management, supported by policies, laws, and investments that help guide advances. It is likely that similar comprehensive policies will be needed and continually adapted to manage open-ended design processes for synthetic biology safely.

In “The Origins of Order” (*188*), Kauffman estimates that a basic set of 10^5^ to 10^8^ proteins, a large but not insurmountable number, would suffice to engineer any biochemical transformation rapidly. Three decades later, the synthetic biology community can dream of being even more ambitious, establishing a strategy to realize any biological function we might need? Creativity will surely be a key component in meeting this goal, and novelty is the fuel required to power it.
